# Potential value and chemical characterization of gut microbiota derived nitrogen containing metabolites in feces from *Periplaneta americana* (L.) at different growth stages

**DOI:** 10.1038/s41598-021-00182-0

**Published:** 2021-10-27

**Authors:** Weiqi Lv, Ying Cui, Gen Xue, Ziyan Wang, Lu Niu, Xin Chai, Yuefei Wang

**Affiliations:** State Key Laboratory of Component-Based Chinese Medicine, Tianjin Key Laboratory of TCM Chemistry and Analysis, Tianjin University of Traditional Chinese Medicine, Tianjin, 301617 China

**Keywords:** Biochemistry, Computational biology and bioinformatics, Drug discovery, Zoology

## Abstract

The American cockroach, *Periplaneta americana* (L.), is able to highly survive in various complicated environments around the globe, and often considered as a pest. In contrast, billions of *P. americana* have been massively reared in China and extensively used as a medicinal insect, due to its function for preventing and treating ulceration and heart failure. Considering the possibility that microbiota-derived metabolites could be an effective source to identify promising candidate drugs, we attempted to establish a rapid method for simultaneous determination of gut microbiota metabolites from medicinal insects. In this study, network pharmacology approach and ultra-performance liquid chromatography (UPLC) technique were employed to reveal the potential pharmacological activity and dynamics variation of nitrogen-containing metabolites (NCMs) originated from the gut microbiota of breeding *P. americana* at different growth stages. A metabolites-targets-diseases network showed that NCMs are likely to treat diseases such as ulceration and cancer. The analysis of NCMs' content with the growth pattern of *P. americana* indicated that the content of NCMs declined with *P. americana* aging. Both principal component analysis and orthogonal partial least squares discriminant analysis suggested that 8-hydroxy-2-quinolinecarboxylic acid and 8-hydroxy-3,4-dihydro-2(1*H*)-quinolinone are the potential differential metabolic markers for discriminating between nymphs and adults of *P. americana*. Moreover, the developed UPLC method showed an excellent linearity (*R*^*2*^ > 0.999), repeatability (RSD < 2.6%), intra- and inter-day precisions (RSD < 2.2%), and recovery (95.5%–99.0%). Collectively, the study provides a valuable strategy for analyzing gut microbiota metabolites from insects and demonstrates the prospects for discovering novel drug candidates from the feces of *P. americana.*

## Introduction

American cockroach, *Periplaneta americana* (L.), is an insect within the family Blattidae, which usually survives in complex and diverse habitats, ranging from the wild forest and mountains to urban environments^1^. It is widely known as the most common household pest that causes nuisance to peoples' homes. In China, it is used as a medicinal ingredient in traditional Chinese medicine (TCM) for treating blood stasis, chills, fever, and intra-abdominal mass, which has been recorded in two famous classical documents^2^, *Shen Nong Ben Cao Jing* and *Compendium of Materia Medica*. Recent studies have proved that *P. americana* is enriched with active compounds, including amino acids, polypeptides, polysaccharides, nucleic acids, lipids, alkaloids, etc^3^. The ethanol extract of *P. americana* exhibits beneficial pharmacological activities, including anti-inflammatory^4^, anti-microbial^5^, anti-tumor^6^, hepatoprotective^7^, cardioprotective^8^, wound healing^9^, and tissue repair promotion effects^10^. In addition, three Chinese patent medicines (Kangfuxin liquid, Ganlong capsule, and Xinmailong injection) have been developed to manage or treat ulceration^11^, hepatic injury^12^, and heart failure^13^, respectively. These medicines prepared from *P. americana* are safe and low toxicity^14^.

Gut microbial metabolites from humans and other creatures are momentous sources for discovering new drugs^15^ and TCMs^16^. The gut microbiota and their metabolites play a significant role in the host's overall health and well-being. Metabolites from normal gut microbiota defend against illness and boost immunity^17^, while the metabolites produced by dysfunctional gut microbiota often cause serious health problems^18^. Before Antony van Leeuwenhoek opened the door to understanding the microbial world in the seventeenth century, Chinese scholars actively utilized metabolites from gut microbiota as therapeutic agents. More than one thousand years ago, herbal doctors had achieved inspiring results by treating difficult miscellaneous diseases with medicinal animal feces. These medicinal feces include Jinzhi originated from human feces^19^, Cansha derived from silkworm feces^20^, and Ambergris produced from cetacean feces^21^, which have been summarized and extensively documented in *Compendium of Materia Medica*. Influenced by food source, digestive system, age, environment, and other factors, gut microbiotas in different organisms are specific about the abundance and structure, resulting in differences in the content and types of metabolites^22^. Modulation of beneficial gut microbiota and their biologically active metabolites have been shown to be a therapeutic way for maintaining human health^23^. As a result, the therapeutic approaches, such as fecal microbiota transplantation, are increasingly becoming attractive against several diseases with the proved clinical efficacy^24^. Moreover, in the light of the importance of gut microbiota in health, researchers have urgently developed diverse strategies and techniques to study gut microbiota structure, composition, and metabolites. Multiple techniques have been developed under genomics^25^, metabolomics^26^, proteomics^27^, and organic chemical analysis^28^. Also, health, disease, and disease-causal relationships among host, host gut microbiota, and gut microbiota metabolites are continually explored in the medical^29^ and biomedical fraternity^30^.

Nowadays, farmers breed *P. americana* on large scales under Good Agricultural Practices (GAP) in different farm sites in southwest China. Nevertheless, the proper disposal of feces from *P. americana* has become an intractable problem. Resource utilization of the feces from *P. americana* will strongly support environmental protection and promote economic profit. Although the gut microbial community of *P. americana* is complex and extensive^31^, its gut microbiota is more stable to dietary shifts than the highly responsive nature of gut microbiota from humans and other mammals^32^. Therefore, several active secondary metabolites produced via gut microbiota metabolism of proteins, lipids, and sugars^33^ are speculated to be the reason for the strong vitality and environmental adaptability of *P. americana*. Bacteroidetes is one of the central microbial phylum communities of *P. americana* gut microbiota^32^, which is identified as the predominant proteolytic bacterial colony in feces^34,35^. Protein, the main ingredient of *P. americana* diet, is the primary source of essential amino acids in *P. americana*. The Blattabacterium, a member of the Bacteroidetes, is known to metabolize proteins into amino acids^36^. The nitrogen-containing metabolites (NCMs) that derived from amino acids, such as alkaloids and biogenic amines, are a great source of biologically active secondary metabolites^37,38^. The NCMs might be the key point to explain various pharmacological activities of *P. americana*. In addition, during development and growth of *P. americana*, changes are observed within its gut microbiota population, resulting in influencing production of metabolites^39^. Up to date, the chemical profiling, pharmacodynamics, and clinic utilization of *P. americana* have been extensively reported by numerous studies^40,41^. Among these studies, most of reports focused on the community composition and structural changes of *P. americana* gut microbiota^42^, while reports were limited on the gut microbiota metabolites from *P. americana*. Similarly, various strategies for detecting metabolites from feces by ultra-performance liquid chromatography-tandem mass spectrometry^43^ and gas chromatography-mass spectrometry^44^ have been reported. Nonetheless, studies on the rapid detection and precise quantitative analysis of NCMs have been rarely reported.

**Figure 1:**
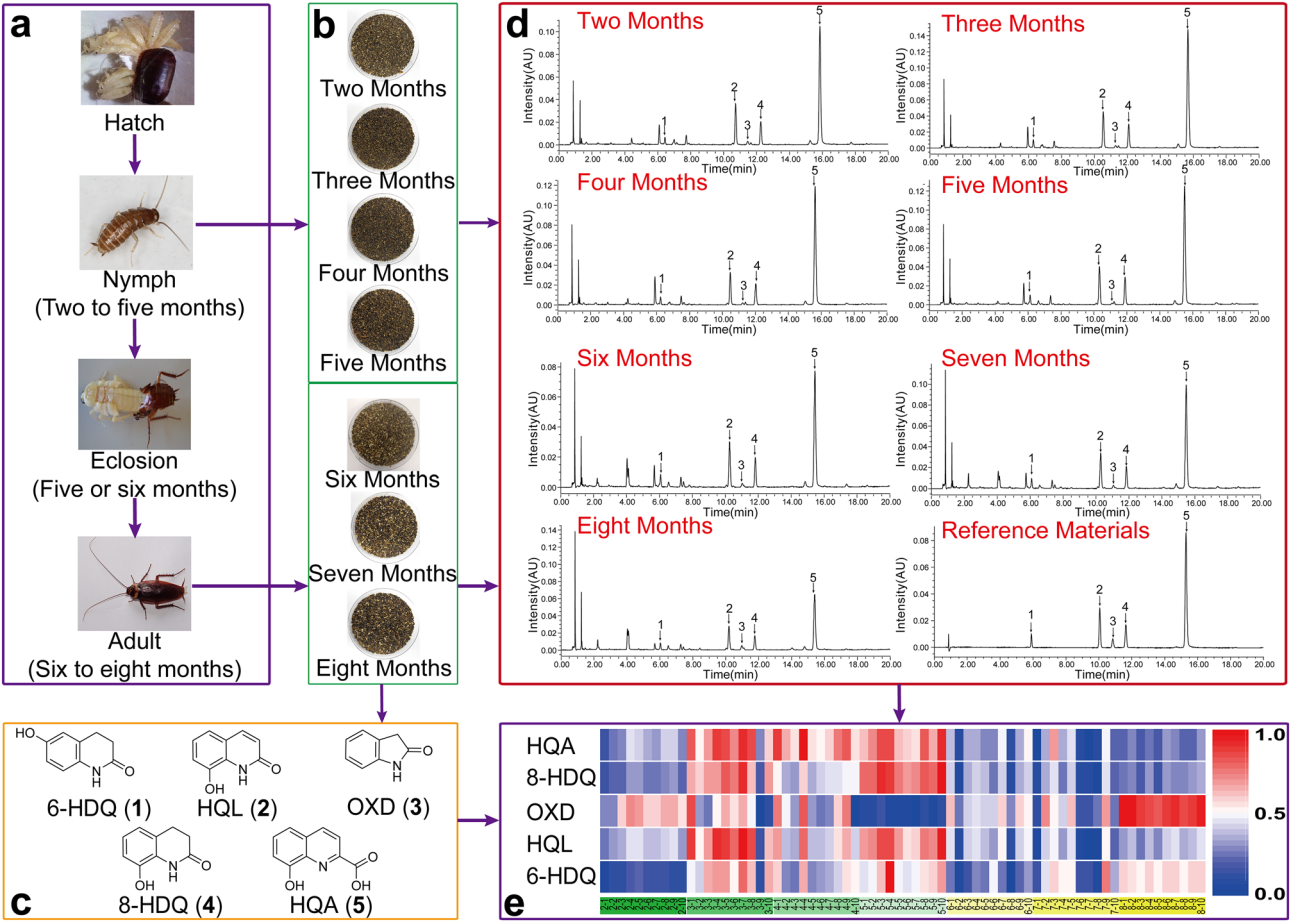
Collection process and the results of fecal samples analysis of *P. americana* at the different growth stages. (**a**) *P. americana* at the different growth stages. (**b**) Collected feces from *P. americana* at 2–8 months. (**c**) Chemical structures of five nitrogen-containing metabolites (NCMs). (**d**) The results of UPLC analysis. (**e**) Heat map for NCMs content in fecal samples. Each column represents one sample and each row represents one of NCMs.

Herein, NCMs (Supplementary Fig. S1 and Fig. 1c) were detected with strong ultraviolet absorption, including 6-hydroxy-3,4-dihydro-1*H*-quinoline-2-one (6-HDQ), 8-hydroxy-2-(1*H*)-quinolinone (HQL), 2-oxindole (OXD), 8-hydroxy-3,4-dihydro-2(1*H*)-quinolinone (8-HDQ), and 8-hydroxy-2-quinolinecarboxylic acid (HQA). These NCMs have been revealed as active compounds responsible for amelioration of diseases through network pharmacology. Accordingly, an ultra-performance liquid chromatography (UPLC) method was established for determining NCMs from *P. americana*. Our results demonstrated that HQA and 8-HDQ are the distinguished metabolic markers for nymphs and adults of *P. americana*. In general, our study proposes a rapid detection method for NCMs and reveals variations of NCMs' content with the different growth stages of *P. americana*.

## Results and discussion

### Exploration of potential pharmacological value of NCMs

The NCMs separated from *P. americana* feces were identified as nitrogen-containing compounds with a benzene ring structure, which were classified to be alkaloids as previously described. This suggests that NCMs might be active compounds with potential therapeutic benefits. Therefore, a network pharmacology approach was used to explore the possible pharmacological activities and action mechanisms of NCMs.

**Figure 2:**
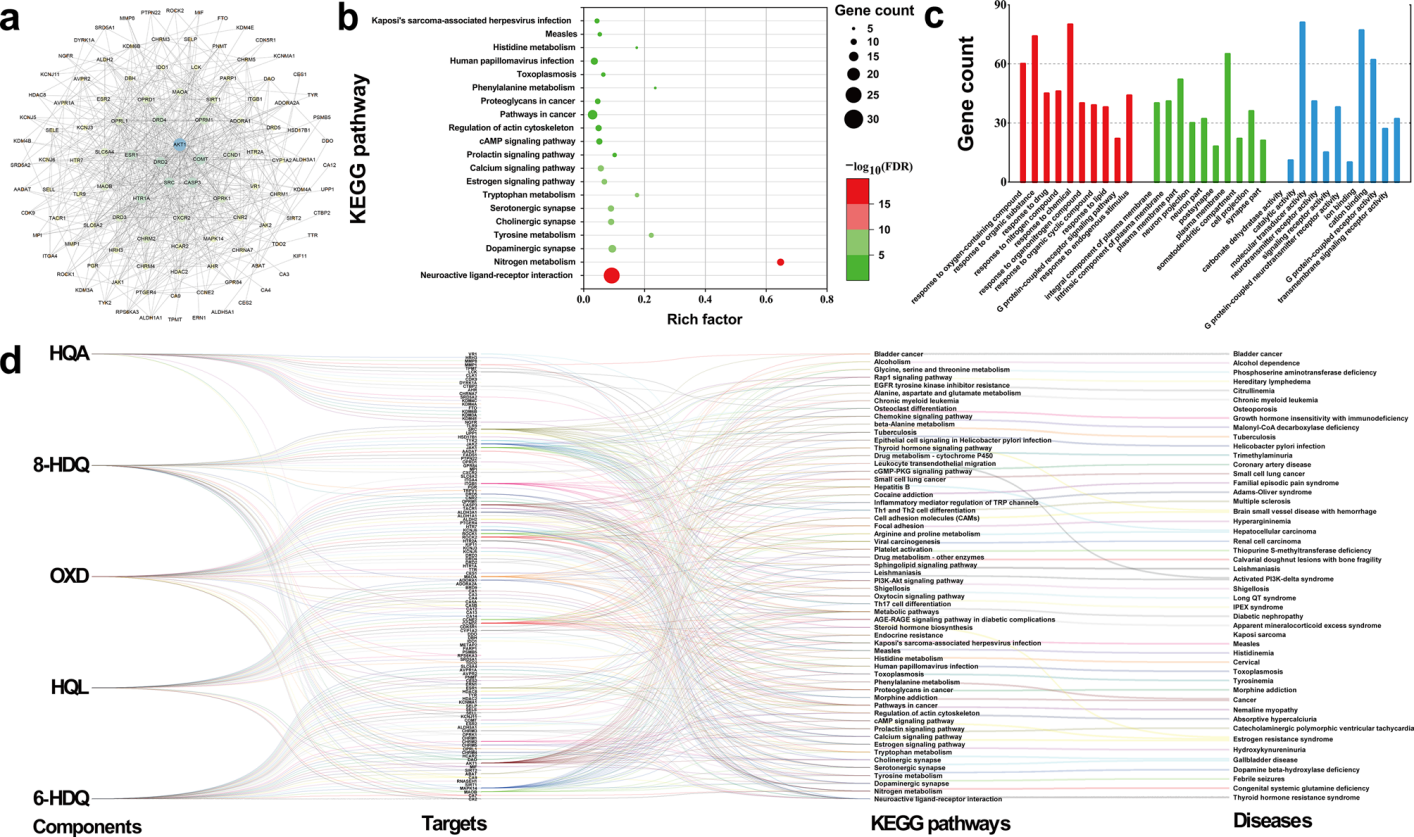
The results of network pharmacology analysis of NCMs. (**a**) Protein–protein interaction (PPI) network for the predicted targets of NCMs. (**b**) Top 20 of Kyoto Encyclopedia of Genes and Genomes (KEGG) pathway enrichment terms for NCMs. (**c**) Gene Ontology (GO) enrichment terms of NCMs involving Biological Process (BP), Cellular Component (CC), and Molecular Function (MF). (**d**) Compounds-targets-pathways-diseases network represents the potential relationships among NCMs, targets, action mechanisms, and diseases.

After eliminating duplicates, 124 targets associated with NCMs were obtained from the Swiss Target Prediction database. Then, the targets were uploaded to the STRING database to investigate the functional partnerships based on protein–protein interactions (PPI) network^45^. After hiding the disconnected nodes, PPI network (Fig. 2a) with a minimum interaction score of 0.4 was imported into Cytoscape software to visualize, which comprised 112 nodes and 570 edges. Moreover, the topological parameters of the node degree, which revealed the number of interactions, were calculated by the network analyzer function of Cytoscape software. Finally, a total of 19 proteins (Table 1) were regarded as key target proteins of the network with the degrees of 20 or higher. Among them, AKT1, SRC, CASP3, COMT, and DRD2 had significantly higher values in degrees, betweenness centrality, and closeness centrality than other proteins in the network, indicating that these proteins are essential in biochemical processes. ATK1, a serine/threonine-specific protein kinase, is the central node of signaling pathways regulating cell survival^46^. In the PPI network, ATK1 was identified as the foremost target. SRC^47^ and CASP3^48^ have been reported as key targets for regulating cell proliferation and apoptosis, while COMT^49^ and DRD2^50^ have crucial roles in dopamine synthesis and secretion.

**Table 1:** The 19 key protein targets of NCMs selected based on three topological parameters, including degree, betweenness, and closeness.

No	Name	Degree	Betweenness	Closeness
1	AKT1	45	0.238	0.587
2	SRC	29	0.076	0.516
3	CASP3	29	0.084	0.496
4	COMT	29	0.079	0.489
5	DRD2	29	0.042	0.489
6	ESR1	27	0.109	0.509
7	HTR1A	27	0.038	0.464
8	DRD4	26	0.024	0.474
9	OPRM1	24	0.023	0.481
10	CCND1	23	0.043	0.472
11	OPRK1	22	0.012	0.472
12	CXCR2	22	0.028	0.451
13	MAOB	21	0.041	0.498
14	DRD3	21	0.007	0.459
15	CHRM2	21	0.009	0.424
16	MAOA	20	0.026	0.470
17	OPRD1	20	0.006	0.430
18	OPRL1	20	0.008	0.464
19	SLC6A4	20	0.012	0.437

The focused potential targets were submitted to STRING for further enrichment analysis. The Kyoto Encyclopedia of Genes and Genomes (KEGG) pathway annotation showed that the potential targets were significantly enriched in 58 pathways (false discovery rate (FDR) ≤ 0.01) (Supplementary Table S1). The top 20 terms with lower FDR are displayed in bubble charts as shown in Fig. 2b, including neuroactive ligand-receptor interaction, nitrogen metabolism, dopaminergic synapse, tyrosine metabolism, cholinergic synapse, and other biologic mechanisms. The FDR and gene counts for each term correspond to color and size scales of the dots. Each FDR of Gene Ontology (GO) enrichment term was calculated (FDR ≤ 0.01) in terms of the order from small to large. Overall, the 124 targets were enriched into 916 terms under the three main categories of Biological Process (BP), Cellular Component (CC), and Molecular Function (MF). The top ten terms of the three main categories are shown in Fig. 2c. They are represented by red, green, and blue bars, respectively. The height of the bars represents the number of genes in each term. Within BP category, response to chemical, response to organic substance, and regulation of biological quality were the most enriched. The enrichment terms of CC category involved cell part, plasma membrane, and plasma membrane part, etc. Binding, catalytic activity, and ion binding were the highly represented categories in MF. The above results help to predict possible pharmacological activity and action mechanisms of NCMs.

The KEGG pathway and GO enrichment analysis showed primary metabolic pathways and biological functions of the potential targets. The constructive compounds-targets-pathways-diseases network shown in Fig. 2d displays the predicted relationship among NCMs, potential targets, KEGG pathways, and related diseases. Collectively, these results indicate that NCMs are worthy of being studied for their potential pharmacological activities.

**Figure 3:**
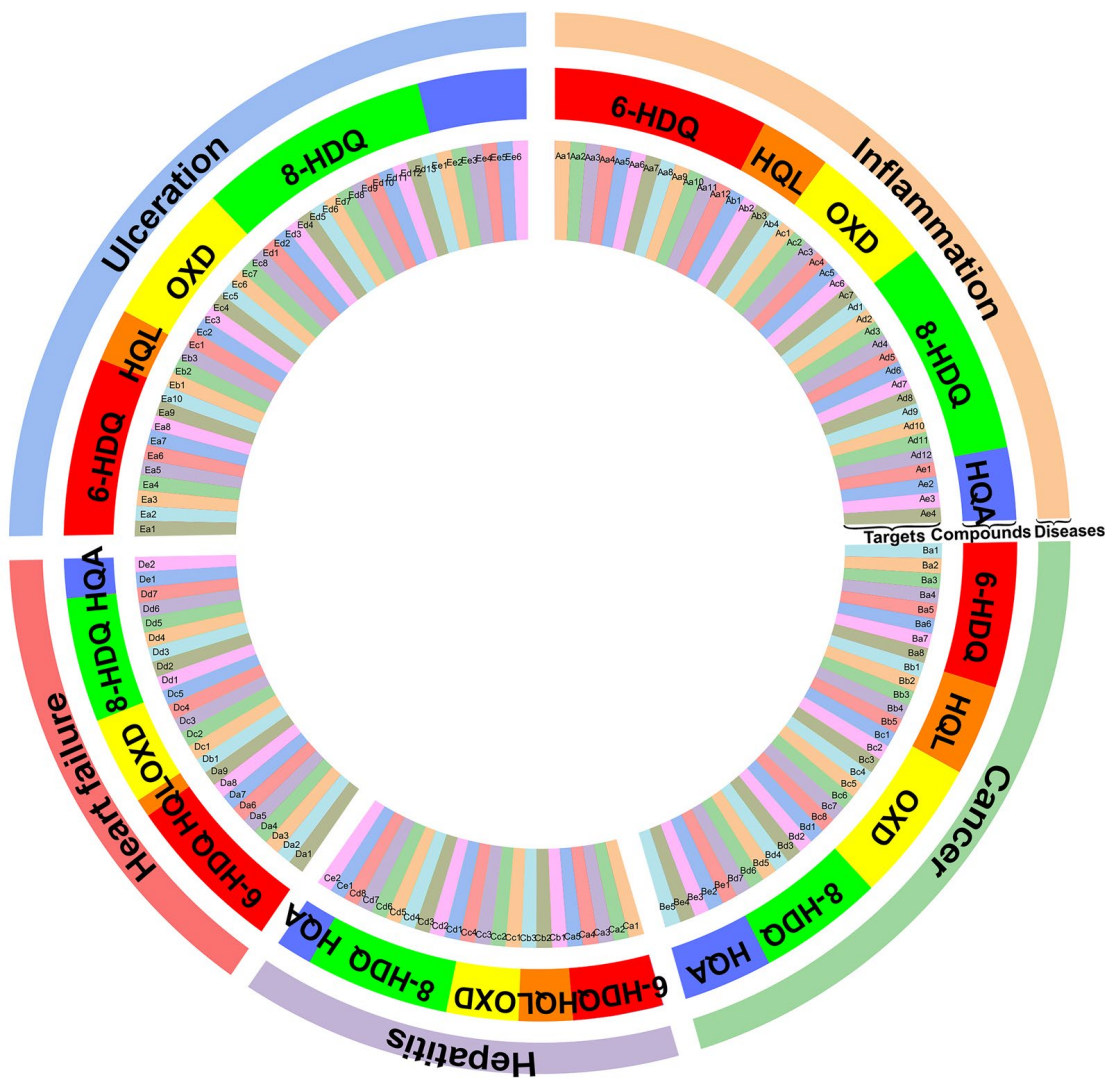
The relationships among *P. americana*-treated diseases (ulceration, heart failure, hepatitis, cancer, and inflammation, shown by the outer ring of doughnut chart), NCMs (6-HDQ, HQL, OXD, 8-HDQ, and HQA, shown by the center ring of doughnut chart) and their intersecting targets (Aa1 to Ee6, shown by the inner ring of doughnut chart).

The doughnut chart (Fig. 3) visualized the predictable relation between the *P. americana*-treated diseases and NCMs. The doughnut chart is composed of three doughnuts that respectively represent *P. americana*-treated diseases (the outer ring of doughnut chart), NCMs (the center ring of doughnut chart), and their intersecting targets (the inner ring of doughnut chart). More intersecting targets (Supplementary Table S2) under the doughnuts mean closer connections between *P. americana*-treated diseases and compounds. This indicates that NCMs are potential active compounds responsible for alleviating diseases such as ulceration and inflammation.

### Optimization of sample extraction and UPLC conditions

Because NCMs have strong ultraviolet absorption, UPLC equipped with UV detector was selected as a suitable analysis strategy. The extraction conditions were optimized to obtain maximum extraction efficiency with minimum expense. The optimization of UPLC conditions was aimed at acquiring better resolution and sensitivity in a shorter time for quantification of NCMs.

Three processing variables, including methanol concentration, solid–liquid ratio, and time of ultrasonic extraction, were tested in the preliminary experiment to determine the effects on extraction yield of NCMs under the different extraction conditions. The levels of three independent variables were investigated as follow: solvents (30%, 50%, and 70% methanol aqueous solution), solid–liquid ratio (1:20 g/mL, 1:50 g/mL, and 1:100 g/mL), and time of ultrasonic extraction (15 min, 30 min, 45 min, and 60 min). The results proved that the optimal extraction conditions of *P. americana* fecal samples were 70% methanol aqueous solution, 1:50 g/mL solid–liquid ratio, and 30 min ultrasonic extraction.

Several UPLC parameters, including mobile phase, column temperature, and detection wavelength, were modified to obtain optimal separation and sensitivity in a shorter time. Addition of formic acid into mobile phase led to better separation and symmetric peak shape. Meanwhile, it has been proved that temperature of chromatographic column is conducive for producing sharper peaks at 50 °C. Chromatographic peaks could be sensitively obtained at 254 nm.

### Methodological validation of UPLC analysis

The linear range, limit of detection (LOD), limit of quantification (LOQ), and determination coefficient of the regression equation of NCMs are shown in Table 2. The results indicate that the calibration curves have satisfactory linearity with determination coefficients from 0.9998 to 0.9999. The LODs and LOQs are in the range of 0.054–0.134 μg/mL and 0.179–0.447 μg/mL, respectively, suggesting that the developed method has high sensitivity. The results of precision, repeatability, stability, and recovery rate are also listed in Table 2. The RSDs of intra- and inter-day precisions are below 2.2%, indicating that the precision of the quantitative method is acceptable. The RSDs of repeatability are all less than 2.6%, suggesting that the method is repeatable enough for the quantitative evaluation of NCMs. The RSDs of stability range from 0.3% to 1.5% within 12 h at room temperature, indicating that NCMs in the sample solutions show high stability. As shown in Table 2, the average recoveries of NCMs range from 95.5% to 99.0%, and RSDs are in the range of 0.4% and 1.7%, revealing that the method has been validated with excellent recovery and accuracy.

**Table 2:** The validation results of the UPLC method including LOD, LOQ, linear range, precision, repeatability, stability, and recovery.

Compounds	LOD (*μ*g/mL)		LOQ (*μ*g/mL)	Linear range (*μ*g/mL)	Determination coefficient (*R*^2^)	Precision (RSD%)		Repeatability (RSD%, *n* = 6)	Stability (RSD%, *n* = 6)	Recovery (*n* = 6)	
						Intra-day (*n* = 6)	Inter-day (*n* = 3)			Recovery (%)	RSD (%)
6-HDQ	0.054		0.179	0.179–10.3	0.9999	0.9	1.0	0.7	0.8	99.0	1.0
HQL	0.077		0.257	0.771–49.3	0.9999	0.2	1.3	0.8	0.3	96.5	0.6
OXD	0.080		0.267	0.267–15.4	0.9999	1.1	0.9	0.6	1.1	97.4	1.1
8-HDQ	0.058		0.193	0.579–37.0	0.9999	0.6	1.0	0.5	0.4	95.5	0.8
HQA	0.134		0.447	1.34–85.8	0.9998	2.2	1.5	2.6	0.8	98.1	1.7

### Content determination and heat map analysis of NCMs from *P. americana* fecal samples

In order to understand the possible variations of the five metabolites of *P. americana* at different growth stages, fecal samples collected at 2–8 months were employed for the study. The validated UPLC method was subsequently applied to obtain the chromatograms and detect NCMs in 70 batches fecal samples of *P. americana*. The representative chromatograms of reference materials and samples from the different stages are successively presented in Fig. 1d.

In order to exhibit differences in the metabolic profiles of *P. americana* feces at the seven growth stages, the content database of NCMs was used to construct a heat map, in which the brighter red color indicated the higher content of metabolites, while the brighter blue color showed the lower content. As shown in Fig. 1e, the relative content of 6-HDQ, HQL, 8-HDQ, and HQA is high in samples collected at 3–5 months but decreased with an increased age. However, sudden increase of OXD and 6-HDQ in their content levels is observed in samples collected at 8 months. Generally, these results imply that high content of NCMs is detected during the nymph stage of *P. americana* than the adult stage, which could be likely attributed to changes occurring in *P. americana* compositional state of gut microbiota as the insect aging. It indicates that the quantification of NCMs is essential for the further investigation of quality control and pharmacodynamics of metabolites from *P. americana* gut microbiota. Therefore, the differential metabolites in samples collected at the different months are worth exploring further.

### Principal components analysis (PCA) and orthogonal partial least squares discriminant analysis (OPLS-DA) of gut microbiota metabolites from *P. americana* at the different growth stages

**Figure 4:**
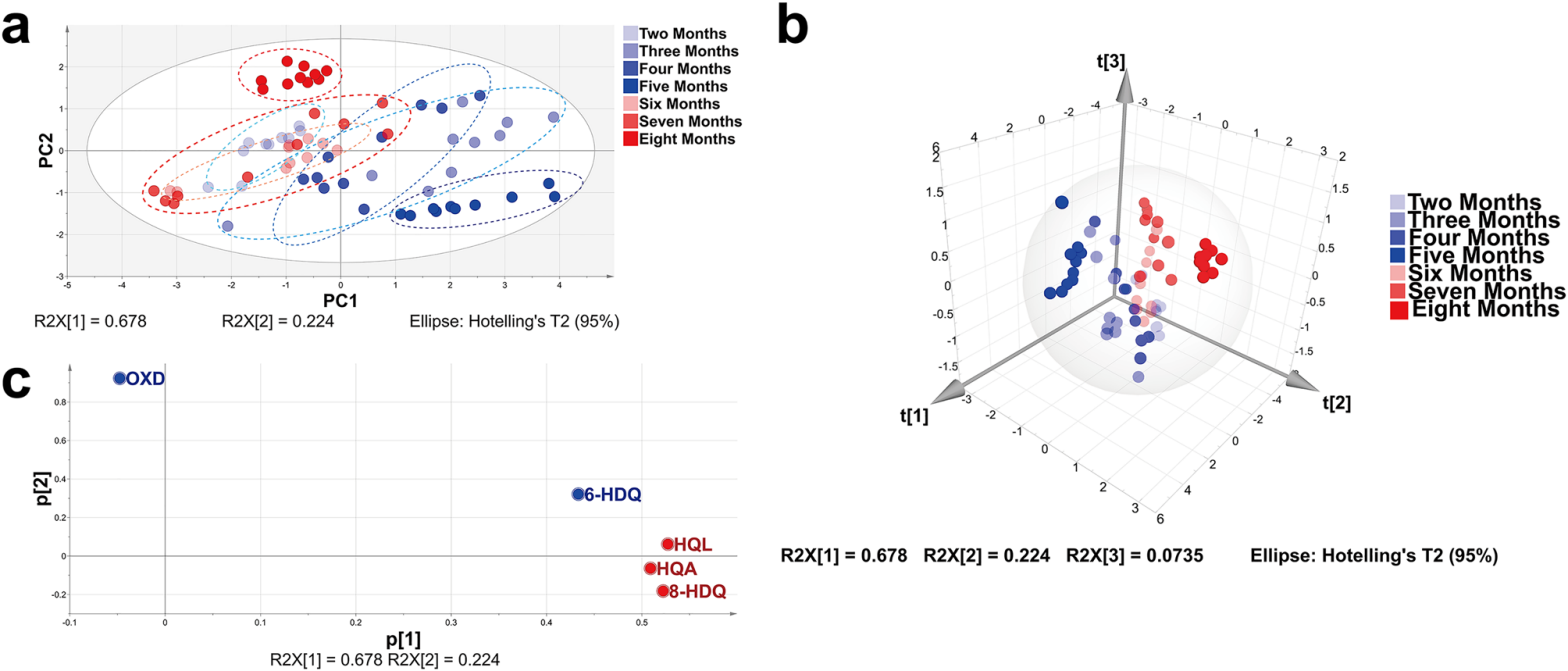
The results of principal components analysis (PCA) for NCMs from *P. americana* at the different growth stages. (**a**) Score plot of PCA model for differentiating samples at the different growth stages. (**b**) 3D score plot of PCA model based on the three principal components. (**c**) Loading score plot from PCA model.

PCA, an unsupervised pattern recognition technique, is used to reduce the dimensionality of the data^51^ and summarize the main characteristics of variables for building predictive models that can be visualized to ascertain relatedness among samples^52^. This tool provides the key information from chemical content data, with a depth overview of clustering patterns. In order to further locate the possible outliers and visualize the potential separation tendency among samples from *P. americana* with the different ages, PCA analysis involving unit variance scaling was performed on the content data of NCMs. The results indicated that the total variance explained by PC1 and PC2 accounted for 90.2% accumulation contribution rate. The PCA score plot (Fig. 4a) showed that the 70 batches samples were divided into seven groups at 95% confidence level without an outlier. All samples except for the samples from 5 and 8 months had intersection areas in the plot, which were not fully separated from the other samples. However, as shown in Fig. 4a, the samples from adult (6–8 months) represented by various shades of red circular dots tend to cluster to the left part, while the samples from nymph (2–5 months) represented by various shades of blue circular dots scatter to the right, depicting that the gut microbial metabolites of nymphs are approximately separated from adults. The three-dimensional (3D) score plot (Fig. 4b) shows the rough spatial separation for nymphs and adults. This separation likely suggests that the gut microbiota compositional state and their dependent metabolic activities change with the aging of *P. americana*. The loading score plot (Fig. 4c) displays that HQL, HQA, and 8-HDQ demonstrate greater contributions in PC1, which are primary factors that influence the separation. Moreover, PCA suggests that NCMs change with growth time, showcasing that NCMs could be used as reference biomarkers for evaluating *P. americana* gut microbiota-dependent metabolites at the different growth stages.

**Figure 5:**
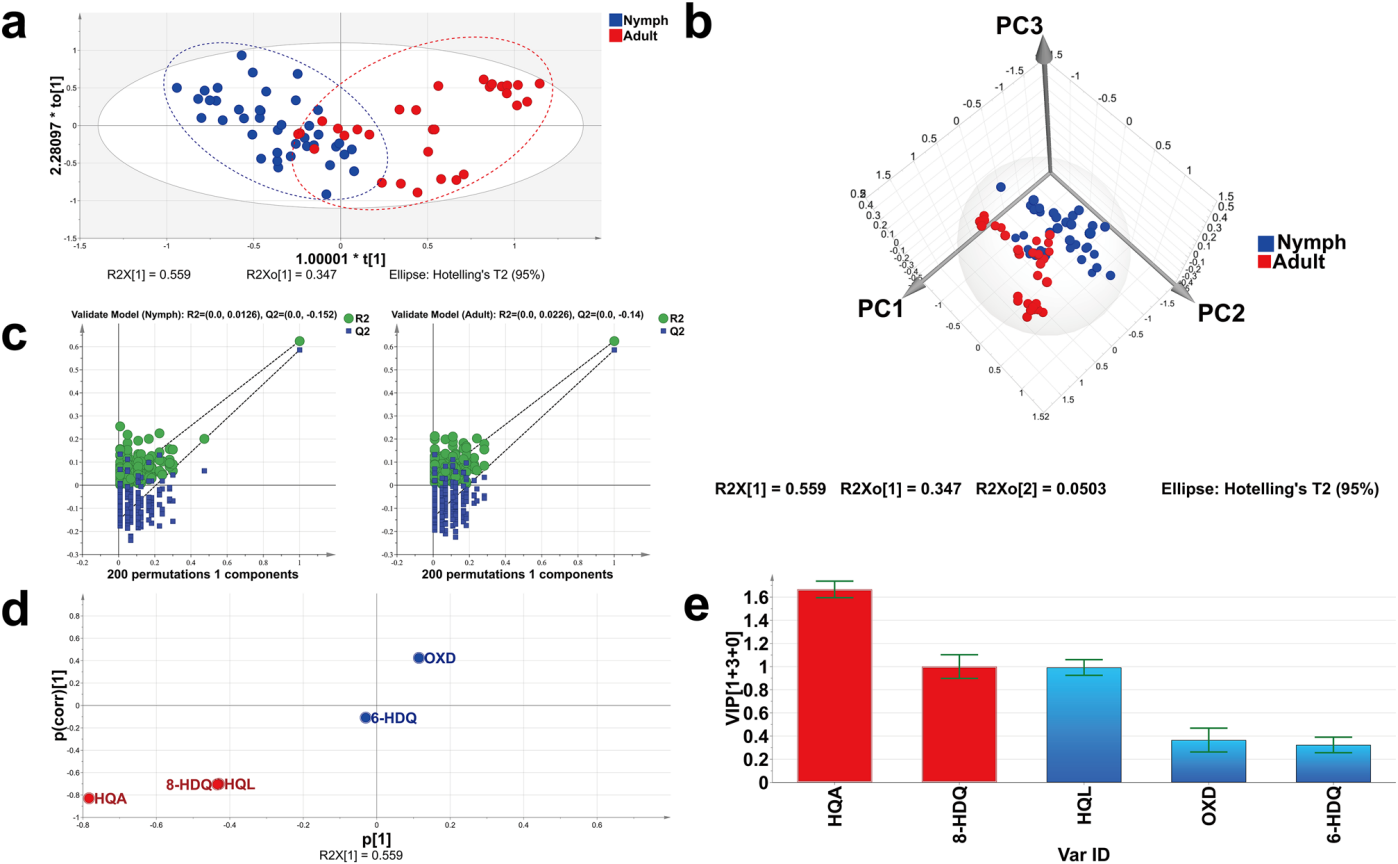
The results of orthogonal partial least squares discriminant analysis (OPLS-DA) for NCMs from nymphs and adults. (**a**) Score plot of OPLS-DA model for differentiating adults from nymphs. (**b**) 3D score plot of OPLS-DA model based on the three principal components. (**c**) Permutation plots of OPLS-DA model with 200 permutation tests. (**d**) S-plot of OPLS-DA model. (**e**) Variable importance for the projection (VIP) of each compound.

The OPLS-DA model, which incorporates an inbuilt orthogonal signal correction filter, can effectively extract principal variables from the known chemical variables to distinguish samples from different sources^53^. Comparing with PCA model, the OPLS-DA model provides a better effective separation performance. In order to find out the different significant metabolites in the feces of nymphs and adults, the quantitative data of NCMs for nymphs aged from 2 to 5 months and adults aged from 6 to 8 months were further analyzed by OPLS-DA. The data matrix was dealt with Pareto (Par) scaling. Compared with PCA model, interpretability (R_cumulated_^2^ = 0.625) and prediction performance (Q^2^_cumulated_ = 0.586) of the OPLS-DA model with one predictive and three orthogonal (1 + 3) components were enhanced. As shown in Fig. 5a, the adult and nymph samples are clustered in the right and left parts of the plot, respectively. An obvious separation between nymphs and adults is also found in 3D score plot (Fig. 5b). The *p*-value (2.599 × 10^–9^) acquired by CV-ANOVA tests is less than 0.05, which testify the predictive capability of this model. After that, the permutation test (*n* = 200) was performed to validate the model performance. As shown in Fig. 5c, the values of R^2^ (= 0.0, 0.0126) and Q^2^ (= 0.0, − 0.152) for nymphs and R^2^ (= 0.0, 0.0226) and Q^2^ (= 0.0, − 0.140) for adults indicate that the OPLS-DA model has a low risk of overfitting. It is manifested that HQA, 8-HDQ, and HQL are the important classification elements as shown in S-plot (Fig. 5d). Also, the two variables (HQA and 8-HDQ) are selected as the differential metabolites with important discrimination capability by virtue of variable importance in projection (VIP) ≥ 1 (Fig. 5e). The result reveals that HQA and 8-HDQ are the distinguished metabolic markers for nymphs and adults. Accordingly, a similar variation tendency for HQA and 8-HDQ in different stages is observed in heat map plots, indicating that the aging of *P. americana* influences the relative amount of HQA and 8-HDQ. Additionally, the two differential metabolites are closely related to ulceration than other diseases, as shown in Fig. 3 and Supplementary Table S2.

## Conclusion

In this study, a network pharmacology approach was devoted to elucidating possible pharmacological activity and action mechanisms of NCMs. Meanwhile, a fast and stable UPLC method was established to simultaneously quantify NCMs from *P. americana* at the different growth stages. Multivariate statistical methods and a network pharmacology approach were used to analyze the differences between nymphs and adults of *P. americana*. Our findings show that HQA and 8-HDQ can be used as the distinguished metabolic markers between nymphs and adults. The research contributes to analyzing metabolites from insects' gut microbiota and provides a theoretical basis for developing drugs related to *P. americana*.

## Materials and methods

### Network pharmacology analysis

In this study, NCMs' connections with potential targets, pathways, and related diseases were explored via a network pharmacology analysis. The potential targets of NCMs were obtained from the Swiss Target Prediction database platform (http://www.swisstargetprediction.ch/). After deletion of repetitive terms, target symbols of each NCM were merged. Subsequently, the focused targets were imported into the STRING database platform (https://string-db.org/) for PPI analysis, GO terms enrichment analysis, and KEGG pathway enrichment analysis. Furthermore, the PPI network was visualized by Cytoscape 3.7.1 software after deletion of disconnected targets. The degrees, betweenness centrality, and closeness centrality, which indicated the topological importance of targets in the PPI network, were analyzed using Network Analyzer function of Cytoscape software. The terms of GO and KEGG pathways were selected by taking FDR into account. Then the related diseases associated with the filtered KEGG pathways were collected by KEGG database platform (https://www.kegg.jp/kegg/)^54^. The GO terms enrichment analysis was carried out to reveal the GO terms, which were most enriched in BP, CC, and MF. The visualization of KEGG pathways and GO terms were performed by Origin 2019b software (Originlab Corp., Northampton, MA, USA). Finally, with the obtained data, Origin software was used to construct the compounds-targets-pathways-diseases network, displaying the predictable clinical values and action mechanisms of NCMs.

With the notion that NCMs might have similar pharmacological activities to the extracts of *P. americana*, we tried to explore the relationship between NCMs and *P. americana*-treated diseases. Five *P. americana*-treated diseases (including ulceration, heart failure, hepatitis, cancer, and inflammation) were chosen to collect their related targets, which were obtained by capturing the top 1000 closely relevant disease targets exported from GeneCards database platform (https://www.genecards.org/). The targets of NCMs were obtained from the Swiss Target Prediction database platform as described above. The intersection between targets of NCMs and *P. americana*-treated diseases was performed by Venn platform (https://bioinfogp.cnb.csic.es/tools/venny/) and visualized by a form of chart composed of three doughnuts. It was tried to expound the relational degrees between NCMs and the treated diseases by counting intersecting targets.

### Materials and reagents

The fecal samples of *P. americana*, a gift from Yunnan Tengchong Pharmaceutical Co., Ltd, were collected from Stone Mountain GAP breeding base of *P. americana*. *P. americana* was raised strictly according to the GAP standards, and their main diet was soybean powder. Fecal samples of 70 batches were collected at seven growth stages (including 2, 3, 4, 5, 6, 7, and 8 months), and ten batches of each growth stage were collected from the different workshops, which were numbered from 2–1 ~ 2–10 to 8–1 ~ 8–10. The samples collected are shown in Fig. 1a and b. All samples were stored at − 80 °C.

As shown in Fig. 1c, five NCMs including 6-HDQ, HQL, OXD, 8-HDQ, and HQA were obtained with high purity (≥ 95%) from the feces of *P. americana* by our laboratory. Methanol, acetonitrile, and formic acid were purchased from Sigma-Aldrich Company (St. Louis, MO, USA). Ultrapure water was supplied by a Milli-Q system (Millipore, Billerica, MA, USA). All chemicals were of chromatographic grade.

### Preparation of standard solution

The NCMs were used as the reference materials and accurately weighed. 6-HDQ and HQL were respectively dissolved in 70% methanol aqueous solution (*v*/*v*) to obtain their stock solutions (0.515 and 0.548 mg/mL). Similarly, OXD, 8-HDQ, and HQA were dissolved in 30% methanol aqueous solution to prepare their stock solutions at the concentrations of 0.513, 0.529, and 1.072 mg/mL, respectively. Afterward, the five stock solutions were mixed and diluted with 30% methanol aqueous solution to prepare the mixed stock solution, whose concentrations were 10.3 μg/mL for 6-HDQ, 49.3 μg/mL for HQL, 15.4 μg/mL for OXD, 37.0 μg/mL for 8-HDQ, and 85.8 μg/mL for HQA, respectively. All reference solutions were stored at 4 °C before determination.

### Preparation of sample solution

The powdered sample (0.5 g) was transfered into 25 mL volumetric flask and ultrasonically extracted by 70% methanol aqueous solution (540 W, 35 kHz) for 30 min at room temperature. The extracted solution was adjusted to the scale by adding 70% methanol aqueous solution. An aliquot (2.0 mL) of each sample was centrifuged at 14,000 rpm for 10 min, and then the supernatant was filtered through 0.45 mm PTFE membranes. An aliquot (1.0 mL) was diluted with 1.0 mL ultrapure water and mixed. All the samples were carried out in triplicates.

### UPLC analysis

All samples were analyzed on a Waters ACQUITY UPLC system equipped with a PDA detector (Waters, Milford, MA, USA). The mobile phase system was composed of 0.1% formic acid in water (A) and acetonitrile (B). Samples were separated on a Waters ACQUITY UPLC® HSS T3 chromatographic column (2.1 × 100 mm, 1.8 μm, Waters). The gradient program was performed as follows: 0–5 min, 98%–93% A; 5–9 min, 93%–90% A; 9–19 min, 90%–78% A; and 19–20 min, 78%–98% A. The injection volume was 2 *μ*L and the flow rate was maintained at 0.3 mL/min. The column temperature was set at 50 °C and the detection wavelength was 254 nm.

### Methodological validation of the quantitative analysis

Series of seven calibration solutions were prepared to construct regression equations, including 0.179–10.3 μg/mL for 6-HDQ, 0.771–49.3 μg/mL for HQL, 0.267–15.4 μg/mL for OXD, 0.579–37.0 μg/mL for 8-HDQ, and 1.34–85.8 μg/mL for HQA. Calibration curves were constructed by plotting the peak areas versus the corresponding concentration of the tested compounds. The slopes, intercepts, and determination coefficients of the calibration curves were calculated by linear regression analysis. The sensitivity was expressed by LOD and LOQ for the tested compounds. In general, the LODs and LOQs were determined at a signal–noise ratio (S/N) of about 3 and 10 by serially diluting the calibration solution. The intra- and inter-day precisions were calculated by determining the selected sample in six replicates during a single day and by duplicating the experiments for three consecutive days. The repeatability was described by analyzing six samples from the same batch using the same method. The stability was also investigated at 0, 2, 4, 6, 8, 10, and 12 h after initial storage at room temperature. The precision, repeatability, and stability were expressed by RSD. The recovery was validated by adding the standard solution to six parallel sample solutions with known concentrations previously. The mixture was analyzed by the normal method and performed in six replicates. The recovery percentages and RSD were calculated by the formula: Recovery (%) = (Detected amount – Original amount)/Spiked amount × 100%, RSD (%) = (SD/mean) × 100%.

### Multivariate statistical analysis

Data matrix composed of 70 observations and five variables were constructed and analyzed by multivariate statistical analysis. The observations and variables severally represented samples and NCMs. The preprocessing of data, the calculation of parameters, and the quantitative analysis of NCMs were carried out with Microsoft Excel (Microsoft Corp., Redmond, WA, USA). The heat map was used to describe the variations in content, and the visual image was exported by Origin software. SIMCA-P 14.1 software (Umetrics, Umea, Sweden) was used for multivariate analysis, including PCA and OPLS-DA.

## Supplementary Information

Supplementary Information.

## Data Availability

The datasets used and/or analyzed during the current study are available from the corresponding author upon reasonable request.
