# Supplementary material for: Potential value and chemical characterization of gut microbiota derived nitrogen containing metabolites in feces from Periplaneta americana (L.) at different growth stages
Source: Sci Rep. 2021 Oct 27;11:21191. doi: 10.1038/s41598-021-00182-0 (PMC8551289; doi:10.1038/s41598-021-00182-0)
Supplement: Supplementary file 1 — Supplementary Information. [file 41598_2021_182_MOESM1_ESM.pdf]

Potential value and chemical characterization of gut microbiota derived nitrogen containing metabolites in feces from *Periplaneta americana* (L.) at different growth stages

Weiqi Lv, Ying Cui, Gen Xue, Ziyan Wang, Lu Niu, Xin Chai✉, Yuefei Wang✉

State Key Laboratory of Component-based Chinese Medicine, Tianjin Key Laboratory of TCM Chemistry and Analysis, Tianjin University of Traditional Chinese Medicine, Tianjin 301617, China.

These authors contributed equally: Weiqi Lv and Ying Cui.

✉email: chaix0622@tjutcm.edu.cn; wangyf0622@tjutcm.edu.cn

Supplementary information

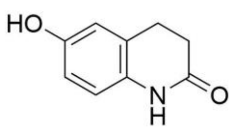

6-Hydroxy-3,4-dihydro-1*H*-quinoline-2-one
(**1**)

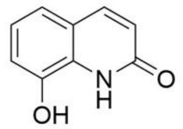

8-Hydroxy-2-(1*H*)-quinolinone
(**2**)

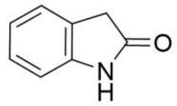

2-Oxindole
(**3**)

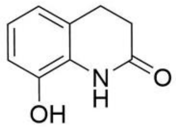

8-Hydroxy-3,4-dihydro-2(1*H*)-quinolinone
(**4**)

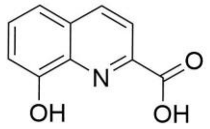

8-Hydroxy-2-quinolinecarboxylic acid
(**5**)

Figure S1. The chemical structures of NCMs investigated in this study.

Table S1. The 58 NCMs-related KEGG pathways including terms, gene counts, and FDR.

Term	Gene count	FDR	Term	Gene count	FDR
Neuroactive ligand-receptor interaction	25	6.18E-19	Metabolic pathways	20	0.00092
Nitrogen metabolism	11	8.28E-16	Platelet activation	6	0.00120
Dopaminergic synapse	12	5.99E-09	Viral carcinogenesis	7	0.00140
Tyrosine metabolism	8	1.55E-08	Arginine and proline metabolism	4	0.00200
Cholinergic synapse	10	2.05E-07	Focal adhesion	7	0.00200
Serotonergic synapse	10	2.05E-07	Th1 and Th2 cell differentiation	5	0.00200
Tryptophan metabolism	7	5.19E-07	Inflammatory mediator regulation of TRP channels	5	0.00210
Estrogen signaling pathway	9	7.72E-06	Cocaine addiction	4	0.00210
Calcium signaling pathway	10	7.91E-06	Morphine addiction	5	0.00210
Prolactin signaling pathway	7	1.09E-05	Hepatitis B	6	0.00210
cAMP signaling pathway	10	1.37E-05	Small cell lung cancer	5	0.00210
Regulation of actin cytoskeleton	10	1.93E-05	cGMP-PKG signaling pathway	6	0.00340
Pathways in cancer	15	2.06E-05	Leukocyte transendothelial migration	5	0.00450
Proteoglycans in cancer	9	8.99E-05	Tuberculosis	6	0.00460
Phenylalanine metabolism	4	0.00011	Drug metabolism - cytochrome P450	4	0.00480
Toxoplasmosis	7	0.00011	Thyroid hormone signaling pathway	5	0.00480
Human papillomavirus infection	11	0.00011	Epithelial cell signaling in Helicobacter pylori infection	4	0.00480
Histidine metabolism	4	0.00028	Chemokine signaling pathway	6	0.00550
Measles	7	0.00032	beta-Alanine metabolism	3	0.00570
Kaposi's sarcoma-associated herpesvirus infection	8	0.00032	Osteoclast differentiation	5	0.00590
Endocrine resistance	6	0.00044	Drug metabolism - other enzymes	4	0.00700
Steroid hormone biosynthesis	5	0.00047	Chronic myeloid leukemia	4	0.00700
AGE-RAGE signaling pathway in diabetic complications	6	0.00047	Alanine, aspartate and glutamate metabolism	3	0.00720
Oxytocin signaling pathway	7	0.00053	EGFR tyrosine kinase inhibitor resistance	4	0.00730

Th17 cell differentiation	6	0.00054	Rap1 signaling pathway	6	0.00840
Shigellosis	5	0.00057	Cell adhesion molecules (CAMs)	5	0.00860
PI3K-Akt signaling pathway	10	0.00072	Glycine, serine and threonine metabolism	3	0.00900
Leishmaniasis	5	0.00085	Alcoholism	5	0.00900
Sphingolipid signaling pathway	6	0.00091	Bladder cancer	3	0.00990

Table S2. The intersecting targets of NCMs related with *P. americana*-treated diseases.

Number	Target	Number	Target	Number	Target	Number	Target
Aa1	MAPK14	Ba2	SIRT1	Cc1	TTR	Ea3	MIF
Aa2	SIRT1	Ba3	CA9	Cc2	ESR1	Ea4	AKT1
Aa3	MIF	Ba4	AKT1	Cc3	ALDH2	Ea5	ESR2
Aa4	AKT1	Ba5	ESR2	Cc4	CASP3	Ea6	SELL
Aa5	COMT	Ba6	COMT	Cd1	MAPK14	Ea7	SELE
Aa6	SELL	Ba7	TYR	Cd2	MPI	Ea8	SELP
Aa7	SELE	Ba8	ESR1	Cd3	PTPN22	Ea9	ESR1
Aa8	SELP	Bb1	RPS6KA3	Cd4	JAK1	Ea10	SLC6A4
Aa9	HDAC2	Bb2	PARP1	Cd5	JAK2	Eb1	MIF
Aa10	TYR	Bb3	CCND1	Cd6	TYK2	Eb2	CYP1A2
Aa11	ESR1	Bb4	CCNE2	Cd7	SRC	Eb3	CCND1
Aa12	SLC6A4	Bb5	CA9	Cd8	TLR9	Ec1	TTR
Ab1	PARP1	Bc1	ESR1	Ce1	TPMT	Ec2	ESR1
Ab2	MIF	Bc2	ROCK1	Ce2	MMP1	Ec3	PTGER4
Ab3	IDO1	Bc3	ALDH2	Da1	MAPK14	Ec4	TACR1
Ab4	CCND1	Bc4	ALDH1A1	Da2	AKT1	Ec5	CASP3
Ac1	TTR	Bc5	CASP3	Da3	COMT	Ec6	TRPV1
Ac2	ESR1	Bc6	PGR	Da4	KCNJ11	Ec7	ITGB1
Ac3	HTR1A	Bc7	ITGB1	Da5	SELE	Ec8	ITGA4
Ac4	TACR1	Bc8	CA9	Da6	SELP	Ed1	MIF
Ac5	CASP3	Bd1	MAPK14	Da7	HDAC8	Ed2	MAPK14
Ac6	TRPV1	Bd2	RPS6KA3	Da8	ESR1	Ed3	TRPV1
Ac7	ITGA4	Bd3	JAK1	Da9	AVPR2	Ed4	CXCR2
Ad1	MIF	Bd4	JAK2	Db1	DBH	Ed5	PTPN22
Ad2	MAPK14	Bd5	HSD17B1	Dc1	TTR	Ed6	SELL
Ad3	TRPV1	Bd6	SRC	Dc2	ESR1	Ed7	SELE
Ad4	CXCR2	Bd7	TLR9	Dc3	KCNJ5	Ed8	SELP
Ad5	PTPN22	Be1	KDM4C	Dc4	CASP3	Ed9	JAK1
Ad6	SELL	Be2	SRD5A2	Dc5	SLC6A2	Ed10	JAK2
Ad7	SELE	Be3	AHR	Dd1	MAPK14	Ed11	TYK2
Ad8	SELP	Be4	CA9	Dd2	PTPN22	Ed12	SRC
Ad9	JAK1	Be5	MMP1	Dd3	SELE	Ed13	TLR9
Ad10	JAK2	Ca1	MAPK14	Dd4	SELP	Ee1	NGFR
Ad11	TYK2	Ca2	SIRT1	Dd5	KCNJ11	Ee2	AHR
Ad12	TLR9	Ca3	AKT1	Dd6	JAK2	Ee3	LCK
Ae1	LCK	Ca4	TYR	Dd7	SRC	Ee4	TPMT
Ae2	TPMT	Ca5	ESR1	De1	FTO	Ee5	MMP1
Ae3	MMP1	Cb1	PARP1	De2	MMP1	Ee6	MMP8
Ae4	MMP8	Cb2	CYP1A2	Ea1	MAPK14	—	—
Ba1	MAPK14	Cb3	CCND1	Ea2	SIRT1	—	—
